# Somatosensory processing is altered in people with hip osteoarthritis compared to controls: A cross-sectional study

**DOI:** 10.1177/20494637261491934

**Published:** 2026-10-10

**Authors:** Andrea B. Mosler, Ebonie K. Rio, Benedict M. Wand, Molly E. Coventry, Gordon S. Waddington, Myles C. Murphy

**Affiliations:** 1 Nutrition and Health Innovation Research Institute, Edith Cowan University, Joondalup, WA, Australia; 2 Centre for Injury Prevention and Pain, Institute for Health Research, The University of Notre Dame, Fremantle, WA, Australia; 3 La Trobe Sport and Exercise Medicine Research Centre, School of Allied Health, Human Services and Sport, 2080 La Trobe University, Bundoora, VIC, Australia; 4 Australian International Olympic Committee Research Centre, 2080 La Trobe University, Bundoora, VIC, Australia; 5 Victorian Institute of Sport, Melbourne, VIC, Australia; 6 School of Health Sciences, 3431 The University of Notre Dame Australia, Fremantle, WA, Australia; 7 Research Institute for Sport and Exercise, 2234 University of Canberra, Bruce, ACT, Australia; 8 Australian Institute of Sport, Bruce, ACT, Australia

**Keywords:** arthralgia, chronic pain, pain threshold, pain measurement, somatosensory disorders

## Abstract

**Background:**

With knee osteoarthritis (OA), measures of mechanical and thermal pain sensitivity relate to both pain severity and treatment outcomes, and lower limb left/right judgement is impaired. However, little is known about these somatosensory variables in people with hip OA.

**Objectives:**

Our cross-sectional study aimed to explore the differences in sensitivity to noxious mechanical and thermal stimuli, and lower limb left/right judgement, in people with hip OA compared to matched controls without hip OA.

**Methods:**

Demographic variables and self-reported hip-related disability were collected using an online survey, while physical function was assessed in person using standardised procedures. Quantitative sensory testing was conducted using mechanical (pressure) and thermal pain thresholds. Left/right judgement was assessed through a lower limb left/right judgement task on an online application. Generalised linear regressions were used to analyse differences between groups and explore associations between the demographic variables and the somatosensory measures.

**Results:**

15 participants with hip osteoarthritis and 15 controls were included. Mean (SD) age was 62.1 (11.2) years, median (IQR) BMI 26.2 (4.4) kg/m^2^, and 21/30 (70%) were female. Pressure pain thresholds were lower in people with hip OA at both the hip (p = .049) and the ipsilateral elbow (p = .046). There were no differences detected between groups for thermal pain thresholds (p > .05). Left/right judgement accuracy was lower in controls compared to people with hip OA (p = .038). Associations between the somatosensory measures and demographic variables (sex, age, and BMI) varied across outcomes.

**Conclusions:**

Increased mechanical, but not thermal, pain sensitivity was found in people with hip OA compared to controls. Demographic variables are associated with somatosensory measures and require consideration in any analyses using these measures.

## Introduction

Osteoarthritis (OA) is characterised by degenerative changes to articular cartilage, subchondral bone change, synovitis, and commonly associated with persistent and disabling pain.^1^ Hip and knee OA, which create the largest personal and healthcare system burden in Australia,^2^ are managed using a combination of conservative, pharmacological, and surgical interventions.^3^ However, each of these management options have limitations in terms of success,^4^ and the burden of hip and knee OA continues to grow.^5^ Therefore, a better understanding of the drivers of pain and disability in people with OA might offer innovative management options.

While the degenerative change of chondral surfaces is considered instrumental to OA diagnosis, only a weak relationship exists between the degree of structural chondral change and pain intensity experienced by the patient.^6,7^ Pain associated with OA is better predicted by a cluster of various factors including; pain sensitivity, inflammatory markers, microRNAs, and psychological factors.^8^ However, these are not the only potential pain intensity predictors, previous studies have also found relationships between pain and disability and other factors such as excessive cortical facilitation^9^ or impaired left/right judgement (also termed laterality discrimination).^10^

Quantitative sensory testing (QST) is an overarching term to describe a variety of tests that assess somatosensory function, including sensitivity to noxious stimuli. Specifically, depending on the equipment available, QST can be utilised to quantify stimulus detection thresholds, pain detection thresholds, pain tolerance, and supra-threshold pain intensities using mechanical, electrical, and thermal modalities.^11^ These measures of pain sensitivity may offer insight into the process contributing to a patient’s pain presentation and inform management choices.

A recent individual participant data systematic review and meta-analysis demonstrated that some measures of pain sensitivity were associated with both pain intensity and disability (i.e., PPT) and some may be able to predict treatment response (i.e., thermal pain threshold).^12^ However, these data were almost exclusively conducted in participants with knee OA. From a recent systematic review,^13^ there is only one case-control study in hip OA exploring whether pain sensitivity, specifically PPTs, is associated with pain intensity and disability.^14^ This study compared people with severe hip OA awaiting surgery with controls and reported between-group differences in the proportion of people with altered sensitivity.^14^ Our own recent research demonstrated that people with hip OA did not exhibit differences in the conditioned pain modulation response when compared to controls,^15^ conflicting with knee OA research findings. Therefore, the neurophysiological responses that are associated with knee OA symptoms may differ from those associated with hip OA symptoms.^13^

Impairments in left/right judgement can also reflect disruption of the sensory and/or motor pathways responsible for the planning and execution of movement or the representation of body schema.^16^ Given the presence of gross impairments in descending neural motor drive found in people with hip and knee OA, it is likely that left/right judgement could be impaired in these patients.^17^ Impaired left-right judgement (accuracy and/or speed) of the knee has been demonstrated in people with knee OA,^10,18^ as well as other visuospatial disruptions,^17^ but it is currently unknown whether this phenomenon is present in people with hip OA.

Therefore, our study aims were to explore whether people with hip OA have impaired somatosensory function in comparison to controls without hip OA as measured by PPT, heat pain threshold (HPT), cold pain threshold (CPT), and left/right judgement. We hypothesised that people with hip OA will have lower thresholds for PPT, HPT, CPT, and less accurate left/right judgement in comparison to controls without hip OA.

## Methods

### Study design

We performed a cross-sectional study between April and December 2024, with all data collected at the University of Canberra laboratory, Australia.

### Ethical considerations

This research study was approved by the Edith Cowan University Human Research Ethics Committee (2023-04335-MURPHY), and cross-institutional approval provided by the University of Canberra Human Research Ethics Committee (Project ID: 13465). All participants provided informed, electronic consent.

### Participants

We aimed to include a sample of 30 participants (n = 15 control participants and n = 15 hip OA). For inclusion in the hip OA group, study participants were required to be greater than 40 years of age and have both a clinical and radiological diagnosis of hip OA. Specifically, this required participants to report activity-related hip joint pain and have either osteophytic change (bony spurring) or joint space narrowing on x-ray (as per the American Rheumatology Association criteria^19^). We included participants with either unilateral or bilateral OA, but the more painful side was used for reporting. Controls were age matched and required to be without any confounding pain or lower limb injuries (e.g., ankle fracture) or illnesses restricting their lower limb function.

Participants were excluded if they had been diagnosed with inflammatory polyarthropathy, a centrally driven pain condition (e.g., fibromyalgia), a neurological condition that affects sensory function (e.g., multiple sclerosis and stroke), or reported morning stiffness that lasted greater than 30 minutes. Participants in either group were excluded if they had contraindications for thermal pain testing, for example: cold urticaria; Raynaud’s phenomenon; or any vascular, traumatic, tumoural, infectious, or metabolic lesion of the skin at the site of sensory testing.

### Recruitment

Participants were recruited using promotional flyers via clinical and physical activity networks (e.g., gymnasiums), social media (e.g., closed Facebook groups), and word-of-mouth aiming to include as diverse a sample of participants as possible.^20^ Purposive recruitment of control participants occurred during data collection to optimise age and sex matching.

### Data collection

All included participants completed an online survey administered though the Qualtrics platform, prior to attending the physical assessment session at the University of Canberra in the Australian Capital Territory. The online survey required approximately 30 minutes for completion and all physical assessment sessions were concluded within 75 minutes. For people with hip OA, the affected or most affected side was used for testing. For controls, the side tested was randomly selected via the manual random draw method.

### Data collected via online survey

#### Demographics

The following demographic variables were collected. Age (years); sex (male/female/intersex); gender (man/woman/non-binary/prefer not to say); race, Aboriginal or Torres Strait Islander (yes/no); country of primary residence (self-reported); multi-lingual (yes/no); highest level of education (less than high school degree/high school graduate/bachelor’s degree/master’s degree/doctoral degree/other professional degree (e.g., Medical Doctor); employment status (working full-time/working part-time/student/homemaker or stay-at-home parent/unemployed and looking for work/retired/other); total household income (less than AUD $30,000/between AUD $30,000 and $49,999/between AUD $50,000 and $79,999/between AUD $80,000 and $99,999/between AUD $100,000 and $149,999/between AUD $150,000 and $199,999/more than AUD $200,000/prefer not to say).

#### Hip-related disability

The International Hip Outcome Tool (IHOT-33) is a valid and reliable patient-reported outcome measure and was used to quantify the domains of symptoms and functional limitations; sports and recreational activities; job-related concerns; and social, emotional, and lifestyle concerns.^21^ The Hip and Groin Outcome Score (HAGOS) is a valid and reliable patient-reported outcome measure and was used to quantify the domains of symptoms and stiffness; pain intensity; physical function and daily living; function, sports, and recreational activities; participation in physical activities; and quality of life.^22^ The IHOT-33 and the HAGOS were recommended by the latest international consensus on hip-related pain as the patient-reported outcome measures with the most robust psychometric properties to evaluate hip-related pain and function in young to middle aged active people, thus ideally suited to our cohort.^23^

#### Regions of pain

The Nordic musculoskeletal screen was used to identify regions of aches, pain, and injury in the past 6 months with response options being neck; shoulders; upper back; elbows; wrists/hands; lower back; hips/thighs; knees; and ankles/feet.^24^

#### Psychological status

The 7-item generalised anxiety screen (GAD-7)^25^ and the 9-item patient health questionnaire (PHQ-9)^26^ captured participant anxiety and depressive symptoms, respectively.

#### Physical activity level

Participants reported whether they considered themselves to perform moderate to vigorous physical activity most days (yes/no). Then all participants reported physical activity levels as the number of minutes over the past 7 days engaged in exercise and the intensity level on a rating of perceived exertion scale (0-10). The number of minutes was multiplied by the rating of perceived exertion score to provide an overall figure in arbitrary units that was comparable within and between participant groups.

#### Lifestyle measures

Participants were asked to report their smoking status (yes/no) and alcohol intake (drinks per week).

#### Pain medication usage

Pain medication type and dosage over the previous 7 days was recorded and dichotomised (yes/no) into either taking or not taking medication.

### Data collected via physical assessment

Physical assessment was performed by a single assessor (ABM), who is a highly experienced clinical physiotherapist with over 13 years of research experience. The assessor was not blinded to group allocation (OA vs control).

#### Demographics

The side tested was recorded alongside participant height (cm) and weight (kg), which were used to calculate body mass index (BMI).

### Hip-related pain intensity

The 11-point numerical rating scale of pain (NRS-P 0-10) was used to assess hip pain intensity during a movement known to be provocative for hip OA (sit-to-stand movement).^27^ Seat height was standardised to a set height (50 cm), and participants were provided standardised instructions to report the worst pain intensity they experienced during five sit-to-stand repetitions.

#### Physical function

Each participant’s physical function was evaluated in person using the 30 second chair-stand test and the 40 m fast-paced walk test. These two tests are recommended by the Osteoarthritis Research Society International (OARSI) as core tests to measure physical function for people with hip osteoarthritis.^27^

Sit-to-stand function was performed using a standardised chair without armrests and a seat height of approximately 43 cm. Participants were given standardised instructions to perform the maximum number of chair-stand movements they could in 30 seconds. Ambulatory function, assessed using the 40 m fast-paced walk test, involved timing the participant walking 4x10 m segments with three turns around a cone.^27^ Participants were given standardised instructions to walk the forty m as fast as they could without running.

#### Quantitative sensory testing

One female sex investigator (ABM) conducted all QST assessments of the participants in a temperature- and humidity-controlled laboratory with minimal distractions. Participants were placed on a comfortable bed using pillows to support their elbow and positioned such that the computer monitor was not visible during the testing. Participants were asked to keep their eyes open throughout the session, and sufficient time was given for the participants to accommodate to the position and room and learn about the equipment. Clear explanations were given to participants prior to testing, and identical instructions were delivered to each participant by the single tester (ABM) during the conducting of QST. The test-retest reliability of QST of the lower limb has been reported as fair to excellent in several studies.^28–30^

#### Mechanical sensitivity

Mechanical pressure pain sensitivity was evaluated via PPTs of the painful region (anterior hip) and the non-painful ipsilateral elbow (lateral epicondyle). The anterior hip position was localised and marked as below the inguinal canal at the midpoint between the anterior super iliac spine and the pubis. The PPTs were assessed using a manual algometer with a 1 cm diameter, circular tip. Following the existing recommendations, pressure was applied at a rate of 50 kilopascals (kPa’s) per second.^28,31^

#### Thermal sensitivity

Thermal stimuli were administered using the Medoc Advanced Thermosensory Analyzer TSA2 (Medoc Ltd, Ramat Yishai, Israel) and a 30 × 30 mm (9.0 cm2) CHEPS contact thermode. Uniform skin contact with the thermode was maintained throughout testing using Velcro straps, and the mouse control button was held in the contralateral hand. For safety purposes, temperatures for the heat and cold stimuli were limited to 50°C and 0°C, respectively.

The method-of-limits protocol was followed with computer-controlled delivery of the thermal stimuli. Thermal sensitivity was assessed by measuring heat (HPT) and cold (CPT) pain thresholds by increasing (for HPT) or decreasing (for CPT) the temperature from baseline skin temperature (32°C) at a rate of 1°C per second. Participants were instructed to press the mouse button when the temperature sensation first turned to discomfort. The temperature was then returned to baseline at a rate of 10°C/s. The intertrial interval was 10 seconds, and three trials were performed for both HPT and CPT on the anterior hip and ipsilateral elbow regions. The mean of three experimental trials was accepted as the HPT or CPT. Three trials were selected for the heat and cold pain trials to minimise adaption or sensitisation effects.

#### Left/right judgement

Participants completed a lower limb left/right judgement task using the Neuro Orthopaedic Institute group (Noigroup) recognise application (https://www.noigroup.com/product/recogniseapp/). In the absence of a hip option, the foot application was used to assess left/right judgement. Foot and lower leg images are a commonly used and validated assessment of left/right judgement as they are visually distinctive and provide the variation in orientation and laterality required to assess lower limb left/right judgement.^18^ Images of a left or right lower limb in various postures and orientations were presented on the screen for a maximum of 5 seconds. Participants were seated with legs uncrossed in a room free of auditory or visual distractions. Familiarisation occurred with five images prior to testing, and participants were instructed to indicate on the application if the pictured limb was the left or right as quickly and accurately as possible. The full task was performed once for each participant and accuracy was recorded in the Excel Database.

### Sample size calculations

Between-group sample size was calculated using G.Power 3.1.9.7. Sensory data is typically non-parametric, so we expected to use a Wilcoxon-Mann-Whitney U test to explore between-group differences. Therefore, to detect a large between-group effect size (d > 0.9), using a Wilcoxon-Mann-Whitney U, with a two-tailed approach, α = 0.05, power = 0.80, a total sample of 28 participants was required (14 per group). However, we estimated a portion of participants (∼10%) would be unable to tolerate testing and aimed to recruit a sample of 30 participants.

### Statistical analysis

Data analysis was performed using IBM SPSS Statistics (Version 29.0.0.0). All data were described using frequency and percentage (%) or mean (standard deviation, SD) and median [interquartile range, IQR]. For our primary objective, we performed generalised estimating equations (as data were non-parametric, and clustered by participant) for each of the primary outcomes of interest (PPT, HPT, and CPT) at the hip and the elbow. All models were adjusted for main effects of group, age, sex, and BMI. These covariates were chosen as they have found to be associated with sensory processing in previous research.^12^ For our secondary objective, we also performed generalised estimating equations for left/right judgement accuracy on the tested side. This model was adjusted for main effects of group, age, and sex. Model fit for all analyses were assessed using the Quasi-Likelihood under Independence Model Criterion (QIC). Model residuals were inspected, and all assumptions were met. Statistical significance was accepted when p < .05.

## Results

### Participants

We included 30 participants: 15 with hip OA (73% female sex) and 15 controls (67% female sex). Participants were a mean (SD) age of 62.1 (11.2) years with complete participant descriptives by group provided in Table 1 and Appendix A. All participants lived in Australia and 93% reported their race as being ‘white’ (n = 28). All but one participant were non-smokers (n = 29, 97%) and the median [IQR] number of alcoholic drinks per week consumed by participants were 0 [4]. Most participants reporting being physically active most days (n = 25, 83%), while education level, employment status, and household income were varied (Table 1).

**Table 1.** Participant descriptives.

Variable	Control (*n* = 15)					Hip osteoarthritis (*n* = 15)				
	Mean	SD	Median	IQR	Min to max	Mean	SD	Median	IQR	Min to max
Age (years)	62.0	12.3	60.0	53.0 to 70.0	45.0 to 90.0	62.2	10.5	66	56.0 to 71.0	42.0 to 74.0
Weight (kg)^a^	70.5	15.9	66.9	59.1 to 82.0	44.5 to 100.2	76.6	18	68.5	62.6 to 84.8	55.7 to 120.8
Height (cm)^a^	164.4	12.2	162	154.5 to 173.0	148.5 to 195.0	166.1	7.8	165.5	161.5 to 173.0	153.0 to 181.0
BMI (kg/m^2)a^	25.8	3.2	26.4	24.7 to 28.1	19.4 to 31.0	28.1	7.9	25.9	23.7 to 32.4	18.6 to 49.9
Weekly alcohol intake^a^	3.9	10.2	0.0	0.0 to 4.0	0.0 to 40.0	2.5	4.0	1.0	0.0 to 5.0	0.0 to 15.0
Physical activity (arbitrary units)^a^	2099.1	1664.8	1336.0	675.0 to 3600.0	360.0 to 5775.0	2532.6	2354.5	1730.0	1230.0 to 3180.0	300. to 9360.0
NRS pain with sit to stand (0-10)^a^	0.0	0.0	0.0	0.0 to 0.0	0.0 to 0.0	3.9	1.4	4.0	3.0 to 5.0	2.0 to 7.0
HAGOS: Pain^a^	98.7	94.8	100.0	100.0 to 100.0	20.0 to 100.0	60.8	13.5	60.0	50.0 to 75.0	37.5 to 82.5
HAGOS: Symptoms^a^	97.4	89.9	100.0	100.0 to 100.0	60.7 to 100.0	55.5	11.1	57.1	46.4 to 64.3	35.7 to 71.4
HAGOS: ADL^a^	98.7	94.8	100.0	100.0 to 100.0	80.0 to 100.0	66.0	20.6	65.0	55.0 to 85.0	30.0 to 100.0
HAGOS: Sport^a^	98.5	94.4	100.0	100.0 to 100.0	78.1 to 100.0	51.7	24.9	46.9	37.5 to 65.6	3.1 to 100.0
HAGOS: Physical activity^a^	94.2	77.4	100.0	100.0 to 100.0	12.50 to 100.0	55.0	22.1	62.5	37.5 to 75.0	25.0 to 100.0
HAGOS: Quality of life	98.3	93.5	100.0	100.0 to 100.0	25.0 to 100.0	39.7	18.3	40.0	25.0 to 50.0	10.0 to 75.0
iHOT: Symptoms and functional limitations	72.6	33.6	81.9	62.5 to 100.0	0.0 to 100.0	54.4	15.7	56.3	41.9 to 70.6	30.6 to 78.1
iHOT: Sports and recreational activities	64.2	31.0	66.7	46.7 to 86.7	0.0 to 100.0	41.8	18.2	38.3	31.7 to 56.7	13.3 to 80.0
iHOT: Social, emotional, and lifestyle concerns	55.9	37.3	57.1	14.3 to 90.0	0.0 to 100.0	43.4	21.3	35.7	30.0 to 60.0	10.0 to 82.9
iHOT: Job-related concerns^a^	31.2	41.4	0.0	0.0 to 70.0	0.0 to 100.0	21.8	32.8	0.0	0.0 to 60.0	0.0 to 80.0
Anxiety symptoms^a^	2.7	3.1	2.0	0.0 to 4.0	0.0 to 11.0	5.2	5.1	3.0	1.0 to 8.0	0.0 to 16.0
Depressive symptoms^a^	1.9	2.6	1.5	0.0 to 2.0	0.0 to 10.0	4.3	3.6	2.0	2.0 to 7.0	1.0 to 13.0
30-second sit to stand (reps)	17.4	3.9	18.0	14.0 to 21.0	11.0 to 25.0	16.3	4.7	15.0	12.0 to 20.0	10.0 to 25.0
40m walk test (secs)^a^	25.7	2.7	24.8	23.6 to 27.3	22.0 to 31.8	29.1	8.1	26.5	23.5 to 33.5	21.5 to 47.6

Legend: n = number, SD = standard deviation, IQR = interquartile range, Min = minimum, Max = maximum, BMI = body mass index, NRS = numerical rating scale, Copenhagen Hip and Groin Outcome Score = HAGOS, Activities of Daily Living = ADL, International Hip Outcome Tool = iHOT, 7-item Generalised Anxiety Disorder = GAD-7, 9-item Patient Health Questionnaire = PHQ-9.

a = Data not normally distributed.

## Somatosensory testing outcomes

The median [IQR] for PPTs at the hip was 326.5 [217.8] kPa’s and at the elbow was 281.0 [188.8] kPa’s. The mean (SD) for HPT at the hip was 44.2°C (3.6) and at the elbow was 45.3°C (2.5). The median [IQR] for CPT at the hip was 15.6°C [20.2]and at the elbow was 18.1°C [20.2]. The breakdown by group is detailed in Table 2.

**Table 2.** Descriptive statistics for somatosensory testing by group.

Variable	Control (*n* = 15)					Hip osteoarthritis (*n* = 15)				
	Mean	SD	Median	IQR	Min to max	Mean	SD	Median	IQR	Min to max
Pressure pain threshold: Hip (kPa)^a^	379.9	157.0	371.0	257.0 to 511.0	93.0 to 661.0	290.8	108.6	314.0	202.0 to 347.0	87.0 to 507.0
Pressure pain threshold: Elbow (kPa)^a^	367.8	203.2	355.0	217.0 to 565.0	84.0 to 758.0	257.5	107.3	251.0	186.0 to 344.0	90.0 to 452.0
Heat pain threshold: Hip (°)	44.2	3.9	44.7	41.4 to 48.3	36.9 to 49.2	44.1	3.4	45.0	43.2 to 46.8	36.4 to 47.8
Heat pain threshold: Elbow (°)	45.4	4.2	47.1	45.0 to 48.1	36.8 to 50.0	45.1	4.8	47.0	41.7 to 49.2	35.1 to 50.0
Cold pain threshold: Hip (°)^a^	14.6	11.0	18.7	2.2 to 25.3	0.0 to 27.8	14.9	10.0	9.8	7.8 to 25.2	0.1 to 29.8
Cold pain threshold: Elbow (°)^a^	14.6	10.5	17.5	2.9 to 25.4	0.0 to 25.8	16.8	10.8	21.6	6.0 to 26.1	0.3 to 29.8
L/R judgement: Left (% correct)^a^	87.0	10.0	90.0	80.0 to 100.0	70.0 to 100.0	89.0	10.0	90.0	80.0 to 100.0	70.0 to 100.0
L/R judgement: Right (% correct)^a^	87.0	17.0	90.0	80.0 to 100.0	40.0 to 100.0	91.0	13.0	90.0	90.0 to 100.0	50.0 to 100.0
L/R judgement: Tested side (% correct)^a^	86.0	11.0	90.0	80.0 to 100.0	70.0 to 100.0	92.0	9.0	90.0	90.0 to 100.0	70.0 to 100.0

Legend: secs = seconds, n = number, SD = standard deviation, IQR = interquartile range, Min = minimum, Max = maximum, kPa = kilopascals.

a = Data not normally distributed, L/R = left/right.

### Mechanical sensitivity

Pressure pain thresholds were lower in people with hip OA at both the hip (β = 75.4, 95% CI = 0.26 to 150.6, p = .049) and the elbow (β = 87.6, 95% CI = 1.7 to 173.5, p = .046). Participants with female sex had significantly lower PPTs at both the hip (β = –154.5, 95% CI = –228.3 to −80.7, p < .001) and the elbow (β = –221, 95% CI = –323.2 to −118.7, p < .001). Age and BMI were not associated with PPTs. The complete models are presented in Table 3.

**Table 3.** Generalised estimating equation of pressure pain thresholds at the hip and elbow.

Region	Variable	Beta estimate	95% CI: Lower limit	95% CI: Upper limit	*p*-value
Hip (QIC = 340380)	Group (control)	75.421	0.266	150.577	0.049^a^
	Sex (female)	−154.481	−228.254	−80.708	<0.001^a^
	Age (years)	−2.687	−6.507	1.134	0.168
	Body mass index (kg/m2)	−1.270	−6.556	4.016	0.638
	Intercept	606.853	379.231	834.474	<0.001^a^
Elbow (QIC = 429293)	Group (control)	87.576	1.671	173.480	0.046^a^
	Sex (female)	−220.950	−323.189	−118.711	<0.001^a^
	Age (years)	−1.288	−5.713	3.138	0.569
	Body mass index (kg/m2)	−3.431	−9.382	2.520	0.258
	Intercept	595.880	358.919	832.842	<0.001^a^

Legend: CI = confidence interval, kilopascals, QIC = Quasi-likelihood under the independence model criterion, kg = kilogram, m = metre.

a = statistically significant at p < .05.

### Thermal sensitivity

#### Heat pain thresholds

There were no differences detected in HPT between people with hip OA and controls at either the hip (p = .632) or the elbow (p = .692). A higher BMI was associated with a lower HPT at both the hip (β = –0.22, 95% CI = –0.37 to −0.07, p = .004) and the elbow (β = –0.27, 95% CI = –0.48 to −0.07, p = .010). Female sex was associated with lower HPTs at the elbow (β = -3.778, 95% CI = –6.4 to −1.12, p = .005) but not the hip (p = .057). Age was not associated with HPTs. The complete models are presented in Table 4.

**Table 4.** Generalised estimating equation of heat pain thresholds at the hip and elbow.

Region	Variable	Beta estimate	95% CI: Lower limit	95% CI: Upper limit	*p*-value
Hip (QIC = 304)	Group (control)	−0.521	−2.651	1.609	0.632
	Sex (female)	−2.148	−4.357	0.061	0.057
	Age (years)	−0.034	−0.170	0.102	0.623
	Body mass index (kg/m2)	−0.219	−0.369	−0.068	0.004^a^
	Intercept	53.950	47.099	60.801	<0.001^a^
Elbow (QIC = 406)	Group (control)	−0.521	−3.104	2.062	0.692
	Sex (female)	−3.778	−6.434	−1.121	0.005^a^
	Age (years)	−0.076	−0.224	0.071	0.312
	Body mass index (kg/m2)	−0.273	−0.480	−0.066	0.010^a^
	Intercept	60.248	51.400	69.096	<0.001^a^

Legend: CI = confidence interval, kilopascals, QIC = quasi-likelihood under the independence model criterion, kg = kilogram, m = metre.

a = statistically significant at *p* < .05.

## Cold pain thresholds

There were no differences detected in CPT between people with hip OA and controls at either the hip (p = .740) or the elbow (p = .817). A higher BMI was associated with a warmer CPT at both the hip (p = .028) and the elbow (p = .024). Age and sex were not associated with CPTs. The complete models are presented in Table 5.

**Table 5.** Generalised estimating equation of cold pain thresholds at the hip and elbow.

Region	Variable	Beta estimate	95% CI: Lower limit	95% CI: Upper limit	*p*-value
Hip (QIC = 2980)	Group (control)	1.146	−5.867	8.158	0.749
	Sex (female)	5.130	−2.452	12.713	0.185
	Age (years)	−0.044	−0.387	0.300	0.803
	Body mass index (kg/m2)	0.475	0.053	0.896	0.028^a^
	Intercept	0.540	−20.753	21.833	0.960
Elbow (QIC = 2893)	Group (control)	−0.881	−8.359	6.597	0.817
	Sex (female)	0.963	−7.521	9.448	0.824
	Age (years)	−0.034	−0.351	0.283	0.835
	Body mass index (kg/m2)	0.534	0.072	0.997	0.024^a^
	Intercept	3.173	−18.978	25.323	0.779

Legend: CI = Confidence interval, kilopascals, QIC = quasi-likelihood under the independence model criterion, kg = kilogram, m = metre.

a = statistically significant at p < .05.

### Left/right judgement

Left/right judgement accuracy was significantly lower in controls compared to people with hip OA (p = .038). A higher age was associated with lower accuracy (p = .003). Sex was not associated with judgement accuracy. The complete model is presented in Table 6.

**Table 6.** Generalised estimating equation of left/right judgement.

Variable	Beta estimate	95% CI: Lower limit	95% CI: Upper limit	p-value
Group (control)	−6.441	−12.513	−0.369	0.038^a^
Sex (female)	−5.602	−13.180	1.976	0.147
Age (years)	−0.338	−0.561	−0.115	0.003^a^
Intercept	117.114	103.262	130.967	<0.001^a^

Legend: CI = confidence interval, kilopascals, QIC = quasi-likelihood under the independence model criterion.

a = statistically significant at p < .05.

## Discussion

Our cross-sectional study identified differences in mechanical but not thermal sensitivity in people with hip OA in comparison to controls. Associations between the somatosensory measures and demographic variables (sex, age, and BMI) were found and varied across outcomes. As expected, higher age was associated with lower left/right judgement accuracy, irrespective of group. However, unexpectedly, our participants with hip OA demonstrated better left/right judgement accuracy than controls, which contrasts with previous research findings in knee OA.

Our study found that when using PPTs to quantify pain sensitivity, individuals with hip OA were more sensitive than controls, as evidenced by having lower PPT values at both the hip and the elbow. This finding is consistent with previous reports of reduced PPTs at both local and remote sites in individuals with hip and knee OA.^7,14,32^ We also found that female sex was associated with lower PPTs, consistent with previous findings,^33^ while no relationship was found for age or BMI. Importantly, the observed increase in mechanical sensitivity in our study was apparent at both the testing site local to the pathology (painful hip) and remotely (ipsilateral elbow). These results suggest that central sensitisation, a maladaptive increase in central nervous system responsiveness to noxious and innocuous input, may be a component of the pain and disability seen in people with hip OA.^7,14,34^ Though we cannot disregard that heightened systemic inflammation might contribute to peripheral sensitisation at the elbow in those with hip OA, the absence of thermal sensitivity changes discounts this. Limited evidence suggests that mechanical pain sensitivity, assessed with PPT, has a stronger association with pain severity than radiological severity in hip OA.^7^ However, the relationship between PPT and OA pain intensity and disability requires further exploration as mechanical sensitivity does not appear to predict treatment response.^12^

Differences in thermal sensitivity, as measured by heat or cold pain thresholds, were not observed between hip OA participants and controls in our study. While there is little published evidence examining HPT and CPT in hip OA, our results are consistent with previous research which also showed no difference between these groups.^14^ In contrast, in people with knee OA, there is growing evidence that cold hyperalgesia (measured using CPT) is present, while heat pain thresholds also do not differ with control participants.^35,36^ Our research suggests that sensitisation in hip OA is better assessed using mechanical testing methods (PPT) than thermal; however, further research may be required to draw stronger conclusions.

The finding that people with hip OA performed better with left/right judgement accuracy was not expected given the opposite finding has been reported in other chronic conditions, including knee OA.^10,16^ This difference may be explained by our methodology assessing the foot, in the absence of a hip option. Thus, hip OA participants may be better at left/right recognition in a non-painful body part functionally linked to the painful area, as an adaptation to hip pain. This may be akin to the proprioceptive reweighting that has been demonstrated repeatedly in people with chronic low back pain.^37–39^ In these studies, people with chronic low back pain demonstrate a blunted response to vibratory stimulus delivered to the back muscles, yet a heightened response to vibratory stimulus of the calf. These results may also be explained by the time taken by participants. Specifically, there may be a speed versus accuracy trade off, so whilst people with hip OA might be more accurate, it is possible they could take a lot longer so future research should also record the time to completion. Finally, it could just be that these results represent a type 1 error, and replication of these findings is recommended. Future research could also consider exploring in more detail spatially defined disruptions by assessing motor imagery performance using upper limb images and/or reaction time.^18^

### Limitations

The primary limitation of this study is that it was performed in a single location (The University of Canberra), and whether the participant demographics are generalisable to regions outside of Canberra is unknown. The second limitation to this study is the sample size. While we were powered for our primary outcomes, the relatively small overall sample size (*n* = 30) may mean that we were at greater risk of type 1 and type 2 errors. In particular, we may have been underpowered to detect small associations for thermal sensitivity. We also only recorded accuracy for the left/right judgement task and not reaction time thus potentially limiting the impact of our study findings. Finally, due to the technical nature of the clinical assessment procedures undertaken for our study, the assessor was not blinded to group allocation during the physical assessment, which may have created subconscious measurement bias.

### Conclusion

Our study identified that there are differences in pressure pain thresholds and left/right judgement, but we did not identify thermal sensitivity in people with hip OA compared to controls. To determine if these somatosensory measures have clinical utility, future research should evaluate whether they are associated longitudinally with pain severity or disability or are able to predict treatment outcomes in people with hip OA.

## Supplemental material

Supplemental material - Somatosensory processing is altered in people with hip osteoarthritis compared to controls: a cross-sectional study Supplemental material for somatosensory processing is altered in people with hip osteoarthritis compared to controls: A cross-sectional study by Andrea B. Mosler, Rio Ebonie K, Benedict M. Wand, Molly E. Coventry, Gordon S. Waddington, and Myles C. Murphy in British Journal of Pain.

## Data Availability

Data will be made available on reasonable request.
