# Supplementary material for: Somatosensory processing is altered in people with hip osteoarthritis compared to controls: A cross-sectional study
Source: Br J Pain. 2026 Oct 10:20494637261491934. Online ahead of print. doi: 10.1177/20494637261491934 (PMC13654017; doi:10.1177/20494637261491934)
Supplement: Supplementary file 1 [file sj-pdf-1-bjp-10.1177_20494637261491934.pdf]

**Appendix A. Participant Descriptives**

| Domain | Variables | Control (n) | Hip Osteoarthritis (n) |
|---|---|---|---|
| Sex | Female | 10 | 11 |
| | Male | 5 | 4 |
| Country of Residence | Australia | 15 | 15 |
| Multi-lingual | Yes | 10 | 13 |
| | No | 5 | 2 |
| Race | Asian | 2 | 0 |
| | White | 13 | 15 |
| Aboriginal or Torres Strait Islander | Yes | 1 | 0 |
| | No | 14 | 15 |
| Current Smoker | Yes | 0 | 1 |
| | No | 15 | 14 |
| Taking Pain Medications | Yes | 5 | 7 |
| | No | 10 | 8 |
| Performs moderate to vigorous physical activity most days | Yes | 13 | 12 |
| | No | 2 | 3 |
| Highest level of education | Less than high school degree | 3 | 0 |
| | High school graduate (high school diploma or equivalent including GED) | 2 | 2 |
| | Bachelor's degree | 6 | 7 |
| | Master's degree | 2 | 6 |
| | Doctoral degree | 2 | 0 |
| Current employment | Working full-time | 8 | 4 |
| | Working part-time | 1 | 3 |
| | Student | 0 | 1 |
| | Retired | 6 | 7 |

| | | | |
|---|---|---|---|
| Household income | Less than AUD $30,000 | 1 | 2 |
| | Between AUD $30,000 - $49,999 | 0 | 3 |
| | Between AUD $50,000 - $79,999 | 2 | 2 |
| | Between AUD $80,000 - $99,999 | 2 | 1 |
| | Between AUD $100,000 - $149,999 | 5 | 4 |
| | Between AUD $150,000 - $199,999 | 0 | 2 |
| | More than AUD $200,000 | 5 | 1 |
| Number of Pain Regions | 0 | 1 | 0 |
| | 1 | 4 | 2 |
| | 2 | 5 | 2 |
| | 3 | 2 | 5 |
| | 4 | 1 | 2 |
| | 5 | 2 | 2 |
| | 6 | 0 | 2 |
